# Effect of the TetR family transcriptional regulator Sp1418 on the global metabolic network of *Saccharopolyspora pogona*

**DOI:** 10.1186/s12934-020-01299-z

**Published:** 2020-02-11

**Authors:** Haocheng He, Shuangqin Yuan, Jinjuan Hu, Jianming Chen, Jie Rang, Jianli Tang, Zhudong Liu, Ziyuan Xia, Xuezhi Ding, Shengbiao Hu, Liqiu Xia

**Affiliations:** Hunan Provincial Key Laboratory for Microbial Molecular Biology, State Key Laboratory of Developmental Biology of Freshwater Fish, College of Life Science, Hunan Normal University, Changsha, China

**Keywords:** TetR family transcriptional regulator, Oxidative stress, Butenyl-spinosyn, *Saccharopolyspora pogona*

## Abstract

**Background:**

*Saccharopolyspora pogona* is a prominent industrial strain due to its production of butenyl-spinosyn, a high-quality insecticide against a broad spectrum of insect pests. TetR family proteins are diverse in a tremendous number of microorganisms and some are been researched to have a key role in metabolic regulation. However, specific functions of TetR family proteins in *S. pogona* are yet to characterize.

**Results:**

In the present study, the overexpression of the *tetR*-like gene *sp1418* in *S. pogona* resulted in marked effects on vegetative growth, sporulation, butenyl-spinosyn biosynthesis, and oxidative stress. By using qRT-PCR analysis, mass spectrometry, enzyme activity detection, and *sp1418* knockout verification, we showed that most of these effects could be attributed to the overexpression of Sp1418, which modulated enzymes related to the primary metabolism, oxidative stress and secondary metabolism, and thereby resulted in distinct growth characteristics and an unbalanced supply of precursor monomers for butenyl-spinosyn biosynthesis.

**Conclusion:**

This study revealed the function of Sp1418 and enhanced the understanding of the metabolic network in *S. pogona*, and provided insights into the improvement of secondary metabolite production.

## Background

*Actinomycetes* are gram-positive bacteria that produce a wide array of metabolites. These metabolites have been excavated by genetic engineering technology, and a large number of structurally unique bioactive natural products [1], including antibacterial, anticancer agents, immunosuppressive and anthelminthic agents [2, 3], have been obtained. The genome of *Saccharopolyspora pogona* was sequenced and found to contain a number of gene clusters (GenBank accession no. CP031142), including the spinosyn analogue gene clusters responsible for the biosynthesis of butenyl-spinosyn [4].

As high-quality environmentally friendly biological insecticides, spinosyn analogues have been investigated with different approaches to improve their titres. The heterologous expression is an efficacious method to produce targeted natural products. Zhao et al. found that the heterologous expression of the spinosyn biosynthetic gene cluster was dependent on the expression of rhamnose biosynthesis genes, and with the rhamnose biosynthesis genes expressed under the control of the strong constitutive ermE*p promoter, the titre of spinosyn up to 1 and 1.5 mg/L in *Streptomyces coelicolor* and *Streptomyces lividans*, respectively [5]. Chaoyi Song et al. constructed a spinosyn artificial gene cluster grouped into 7 operons, each with a strong constitutive promoter, and compared with the original gene cluster, the artificial gene cluster resulted in a 328-fold enhanced spinosyn production in *Streptomyces albus* J1074 [6]. Analogously, Tan et al. overexpressed three rate-limiting steps of the heterologous production of spinosyn, and the production of spinosyn was increased gradually and finally reached 1.46 mg/L, which was approximately 1000-fold higher than the level of spinosyn produced by the wild-type strain *S. albus* J1074 [7]. However, probably due to the complex genetic background and the native advantages of natural hosts, the heterologous production of spinosyn analogues usually falls short of expectations. Additionally, The genetic modification is another important strategy for improving the production of secondary metabolites. Jha et al. co-expressed the positive regulators *met*K, *rmb*A, and *rmb*B in *S. spinosa* under the control of the strong ermE* promoter, and the levels of spinosyns A and D in the co-expression strain increased by 7.44/8.03-fold compared with the those in the wild-type strain [8]. The highly conserved protein polynucleotide phosphorylase (Pnp) was considered as a positive regulator, and the overexpression of Pnp notably promoted the butenyl-spinosyn biosynthesis in *S. pogona* [9].

Currently, a substantial number of proteins have been reported to regulate the metabolites of bacteria [10–12], including some global regulators [13, 14], which are of great significance in growth, phenotype and metabolism. TetR family regulators have been reported in numerous organisms [15, 16]; they are involved in the regulating biosynthesis of antibiotics, efflux pumps, osmotic stress, etc., and typically function as repressors [17] to regulate the expression of genes, such as the *ameABC* operon [18] and *fadR* operator [19].

In this study, the *sp1418* (orf02290-1418) gene encoding a TetR family protein was investigated in *S. pogona*, and the present work was motivated by the evidence that the manipulation of *S. pogona* by homologous recombination with an integrative vector resulted in a significant increase in butenyl-spinosyn production and phenotypic changes due to overexpression of the *sp1418*. To gain insight into the molecular mechanisms underlying this phenomenon, we investigated the distinctive proteins of the wild-type strain and mutant strains. The functions of the proteins were analysed, and the results were then validated by functional assays. Evidence indicated that *sp1418* overexpression led to dramatic gene expression changes, which marked affected the growth, butenyl-spinosyn biosynthesis, phenotypes and oxidative stress of *S. pogona*.

## Results

### Sp1418 affects the synthesis of butenyl-spinosyn

**Fig. 1:**
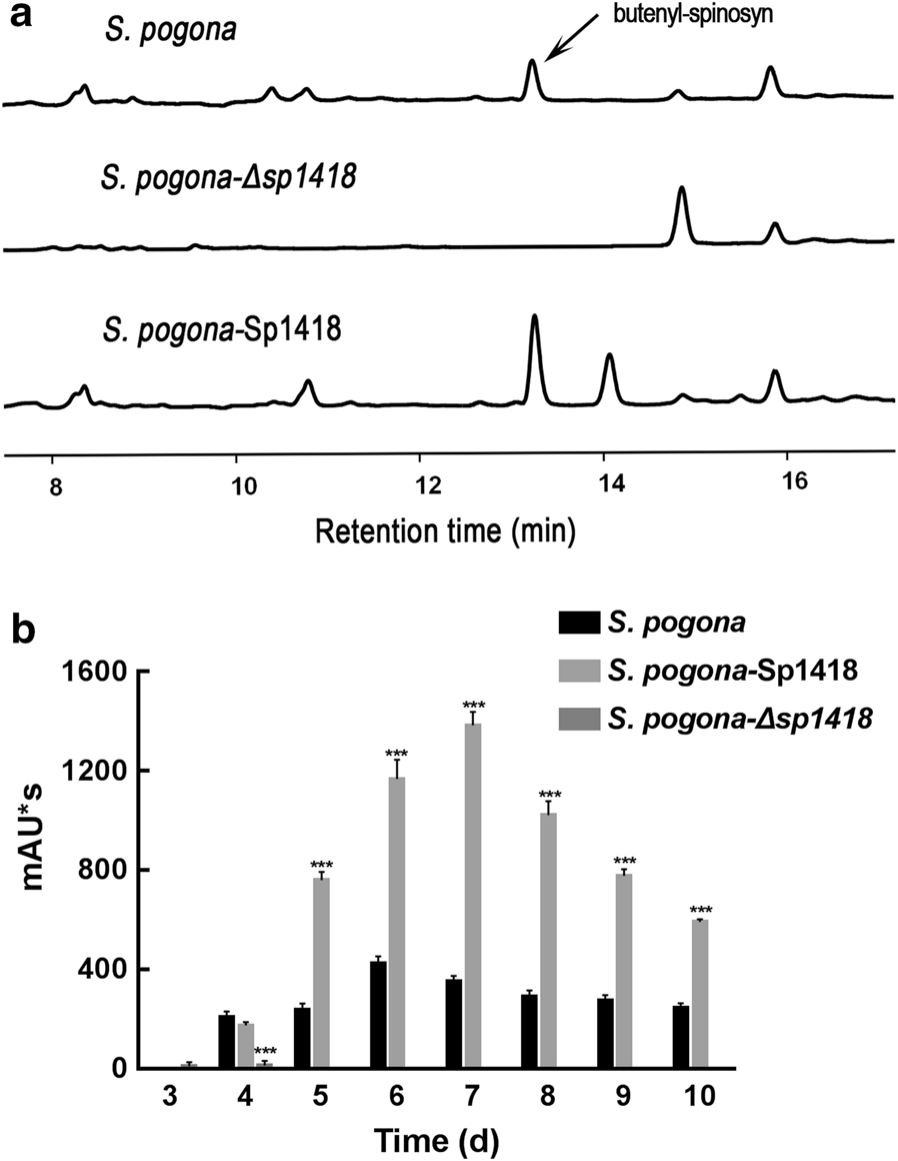
Butenyl-spinosyn production analysis. **a** The HPLC profiles of the wild-type and mutant strains. The detection wavelength was set at 250 nm during the analysis, and the chromatographic peak of butenyl-spinosyn appeared at 13.1 min. **b** Butenyl-spinosyn was detected for 10 days in the wild-type and mutant strains. The maximum production of butenyl-spinosyn in *S. pogona* was 424.3 mAU*s, and that of *S. pogona*-Sp1418 was 1382.9 mAU*s, while butenyl-spinosyn was hardly detected in *S. pogona*-Δ*sp1418* under the same conditions. The maximum concentration of butenyl-spinosyn produced by *S. pogona*-Sp1418 increased by 225.9% as compared to that produced by wild type. *, **and *** indicated P < 0.05, P < 0.01 and P < 0.005, respectively, compared to *S. pogona* under the same conditions. Error bars indicated standard errors of results from n = 3 replicates

**Fig. 2:**
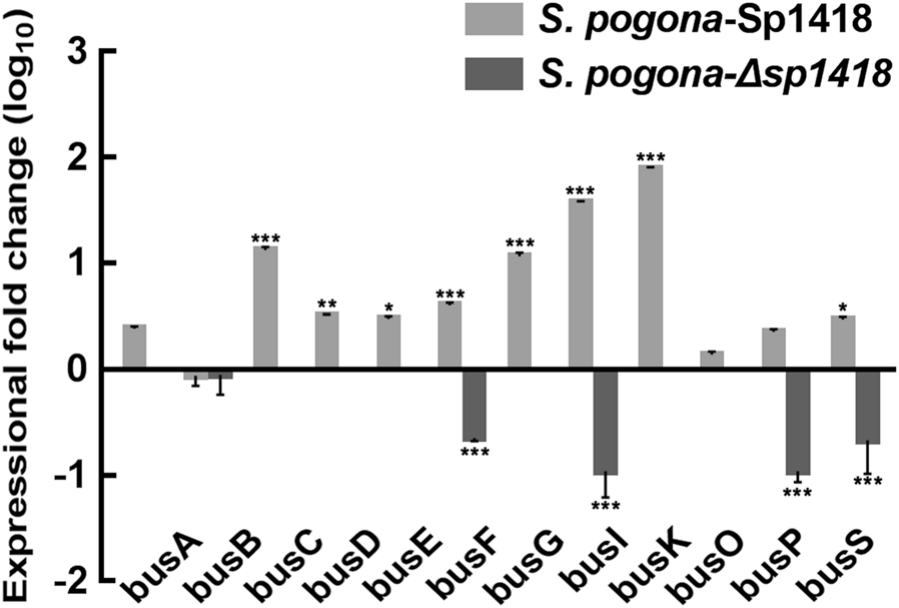
Expression levels of the *bus* genes in *S. pogona* and mutant strains. mRNA samples were isolated from wild-type and engineering strain cells cultured for 144 h, The transcriptional levels (log_10_) of the genes *busA*, *busC*, *busD*, *busE*, *busF*, *busG*, *busI*, *busP*, *busO*, *busK* and *busS* genes in *S. pogona*-Sp1418 were 0.39-, 1.12-, 0.51-, 0.48-, 0.60-, 1.06-, 1.57-, 1.89-, 0.14-, 0.36-, and 0.47-fold higher than those in *S. pogona*, respectively, which indicated a significant upregulation. Limited expression was detected for many *bus* genes in, and only *busB*, *busF*, *busI*, *busP*, and *busS* were detected, with expression levels (log_10_) of 0.05, 0.65, 0.96, 0.96 and 0.67 times that of wild-type bacteria. The 16S rRNA gene was used as an internal control to quantify the relative expression of target genes. Gene expression differences are shown by bar height. Error bars represent the standard deviation of the mean. *, **and *** indicated P < 0.05, P < 0.01 and P < 0.005, respectively, compared to *S. pogona* under the same conditions. Error bars indicated standard errors of results from n = 3 replicates

The butenyl-spinosyns were extracted from the fermentation broth and detected by HPLC (Fig. 1a), and the MS parent ion and characteristic ion data of butenyl-spinosyn component spinosyn αd [20] was confirmed by MS identification (Additional file 1: Figure S1). By detecting the ability of different strains to synthesize butenyl-spinosyn, we revealed that *S. pogona*-Sp1418 began to produce butenyl-spinosyn on the fourth day, and its production was slightly lower than that of *S. pogona.* However, from the fifth day, butenyl-spinosyn production of *S. pogona*-Sp1418 rapidly accumulated and was significantly higher than that of *S. pogona*, and the peak area *S. pogona*-Sp1418 on the seventh day reached a maximum of 1382.9 mAU*s, while that of *S. pogona* was 424.3 mAU*s. The total content of butenyl-spinosyn produced by *S. pogona*-Sp1418 increased by 225.9% compared to that produced by the wild type (Fig. 1b), The butenyl-spinosyn biosynthetic gene cluster consists of 23 *bus* genes, which were responsible for butenyl-spinosyn biosynthesis, and the qRT-PCR results showed that most of the *bus* gene expression levels were significantly upregulated (Fig. 2).

To verify whether this effect was caused by the overexpression of Sp1418, the *sp1418* gene was knocked out in the wild-type bacteria. Only a small amount of butenyl-spinosyn was detected in the knockout bacteria on the third and fourth days, which substantially lost the ability to synthesize butenyl-spinosyn (Fig. 1). The expression of the *bus* genes was severely inhibited. This result implies that the *sp1418* gene could effectively facilitate the butenyl-spinosyn biosynthesis.

### The absence of Sp1418 caused a growth defect and a phenotypic change

**Fig. 3:**
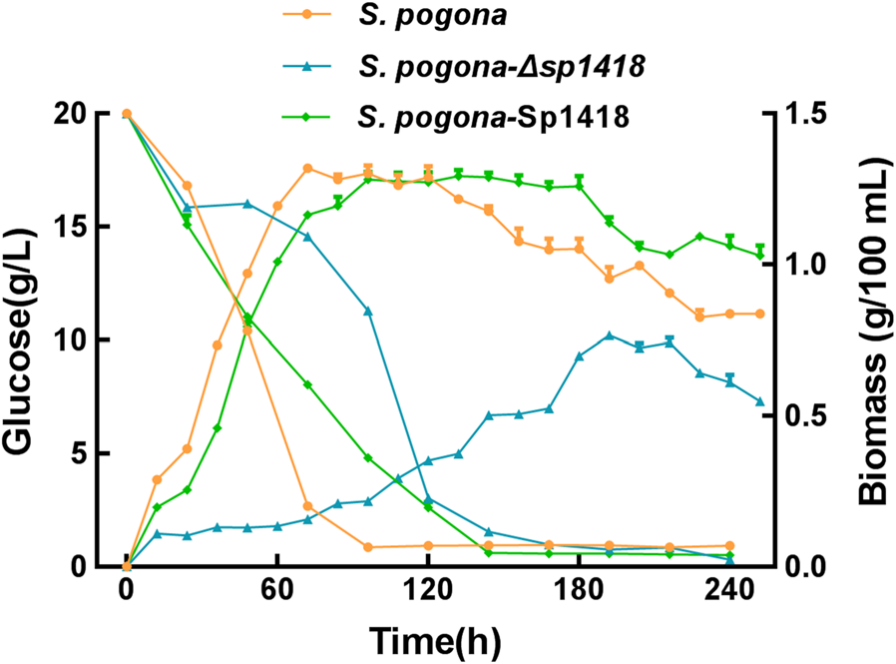
Growth curve and glucose consumption of *S. pogona*, *S. pogona*-Δ*sp1418* and *S. pogona*-Sp1418. *S. pogona*-Δ*sp1418* exhibited a slow growth trend, especially in the logarithmic phase, in which the final biomass values of *S. pogona*-Δ*sp1418* were lower compared to those of wild type and *S. pogona*-Sp1418. *S. pogona*-Sp1418 showed a slightly slower logarithmic growth and entered stationary phase at 96 h, which was 24 h later compared to the wild-type strain, and lasted up to 180 h, which was greatly extended compared to the wild-type strain. Glucose consumption by the knockout strain was the slowest and exhausted in 168 h, which may be related to its growth restriction. The rate of glucose consumption in the logarithmic phase of the overexpression strain was slower, lasting for 48 h more than the wild-type bacteria, and the result was consistent with the growth curve. Error bars indicated standard errors of results from n = 3 replicates

The growth profiles of wild type, *S. pogona*-Δ*sp1418* and *S. pogona*-Sp1418 were significantly different in fermentation broth (Fig. 3). The final biomass values were lower in *S. pogona*-Δ*sp1418* than in wild type, and *S. pogona*-Δ*sp1418* exhibited a slow-growth trend, especially in the logarithmic phase, from 12 to 72 h, the average increase rate of *S. pogona*-Δ*sp1418* biomass was 0.008 g/L/h, which was significantly lower than the wild type’s 0.172 g/L/h. *S. pogona*-Sp1418 showed a slightly delay in logarithmic growth, but the average increase rate of biomass from 12 to 72 h was 0.161 g/L/h, which was comparable to that of the wild-type bacteria, and the stationary phase of *S. pogona*-Sp1418 was greatly extended. The analysis of glucose consumption revealed that the uptake rate of glucose by the knockout strain was 0.027 g/L/h from 24 to 72 h, which was the slowest and may be related to its growth restriction. The uptake rate of glucose from 24 to 72 h of the overexpression bacteria was 0.147 g/L/h, which was lower than the wild type’s 0.295 g/L/h. After 72 h, the glucose of the wild type was basically consumed, but between 72 and 144 h, the glucose content of *S. pogona*-Sp1418 and *S. pogona*-Δ*sp1418* was decreased at a rate of 0.103 and 0.181 g/L/h, respectively.

**Fig. 4:**
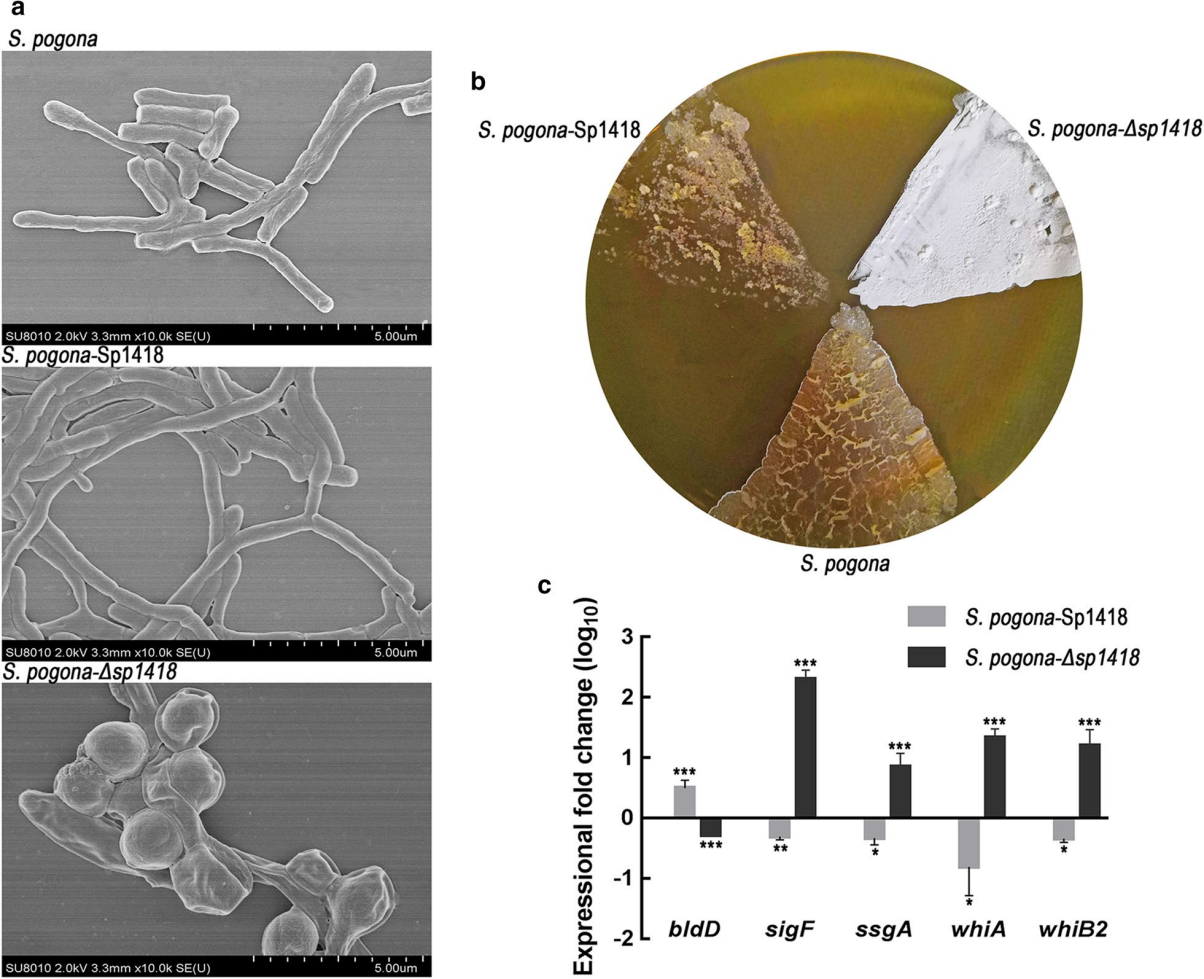
The growth profiles and phenotypic differences in wild type, *S. pogona*-Δ*sp1418* and *S. pogona*-Sp1418. **a** Cold field emission scanning electron micrographs of mycelium. The mycelium of *S. pogona*-Sp1418 grew longer and was more branched than that of *S. pogona.* Little mycelium was found in *S. pogona*-Δ*sp1418*, exhibiting a specific spore-germination phenotype. **b** The phenotypic comparison of the wild-type and engineered strains on CSM medium. Spores were produced on the second day of culture on the medium. *S. pogona*-Δ*sp1418* exhibited a typically white phenotype, which grew more abundantly and faster than the wild-type strain. The spores of *S. pogona*-Sp1418 were hardly observed on the medium. **c** Expression levels of *bldD*, *sigF*, *ssgA*, *whiA* and *whiB* in *S. pogona* and *S. pogona*-Sp1418. mRNA samples were isolated from wild-type and engineered strain cells after 48 h of incubation. The transcriptional levels (log_10_) of the *bldD* gene were 0.49-fold higher in *S. pogona*-Sp1418 and 0.28-fold lower in *S. pogona*-Δ*sp1418* than in *S. pogona*. The transcriptional levels (log_10_) of *sigF*, *ssgA*, *whiA* and *whiB* were 0.31-, 0.33-, 0.81- and 0.34-fold lower in *S. pogona*-Sp1418 than in *S. pogona,* respectively, and were 2.30-, 0.85-, 1.33-, 1.20-fold higher in *S. pogona*-Δ*sp1418* than in *S. pogona*, respectively. The 16S rRNA gene was used as an internal control to quantify the relative expression of the target genes. Gene expression differences are shown by the bar height. Error bars represent the standard deviation of the mean. *, **and *** indicated P < 0.05, P < 0.01 and P < 0.005, respectively, compared to *S. pogona* under the same conditions. Error bars indicated standard errors of results from n = 3 replicates

To observe the cell morphologies, the parental strain and the recombinant strains were grown in CSM broth for 2 days, and the mycelial features of the three strains were observed with scanning electron microscopy (SEM), which revealed that the mycelia of *S. pogona*-Sp1418 were longer and more branched than those of *S. pogona* (Fig. 4a). Moreover, limited mycelium was found in *S. pogona*-Δ*sp1418*, indicating a specific spore-germination phenotype. After being cultured on CSM medium, *S. pogona*-Δ*sp1418* had an obviously white phenotype (Fig. 4b), while the phenotype of *S. pogona*-Sp1418 was fragmented. SEM observations showed that the sporulation ability of *S. pogona*-Δ*sp1418* was improved, and the amount of spores was much more than that of *S. pogona* (Additional file 1: Figure S2).

To explain this phenomenon, we analysed the genes implicated in cell differentiation and mycelial formation in the genome including *bldD*, *whiA*, *whiB*, *ssgA* and *sigF.* The *bldD* is able to regulate a series of downstream sporulation-related genes [21], and positively regulate antibiotic production [22, 23], while the expression of *whiA* or *whiB* is related to the synthesis of the white pigment characteristic of mature *S. venezuelae* spores [24, 25]. In addition, the *ssgA* and *sigF* genes were confirmed to be involved in the regulation of the white phenotype [26–29]. The qRT-PCR analysis revealed that the upregulation and downregulation of these genes were consistent with the expected results (Fig. 4c), indicating that the Sp1418 protein regulates the genes involved in sporulation and phenotype.

### Verification of the *sp1418* expression level

**Fig. 5:**
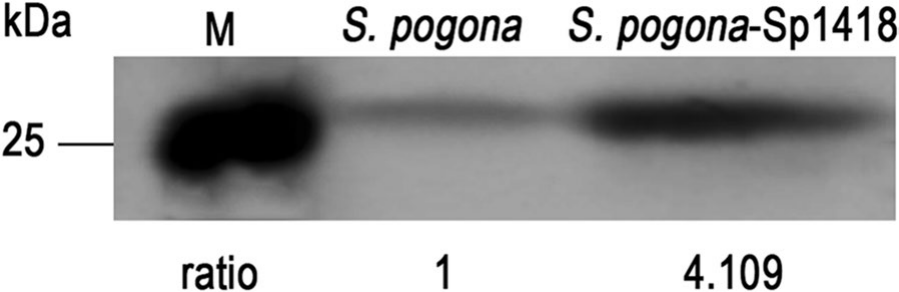
Sp1418 expression level differences between the wild-type and engineered strains. To determine the expression levels of *S. pogona* and *S. pogona*-Sp1418, Western blotting was performed, and the results showed that Sp1418 was 3.11-fold higher in *S. pogona*-Sp1418 than in *S. pogona*

To validate the expression of *sp1418,* a Western blot was performed to show the Sp1418 protein abundance in wild type and *S. pogona*-Sp1418. The heterologously expressed protein Sp1418 (Additional file 1: Figure S3) was identified by 1D-LC–MS/MS (Additional file 2: Table S1), and the anti-Sp1418 antibody was provided by immunizing rabbits. Western blot analysis showed that the Sp1418 expression level was 3.12-fold higher in *S. pogona*-Sp1418 compared to *S. pogona* (Fig. 5).

### Biological activity assay

**Fig. 6:**
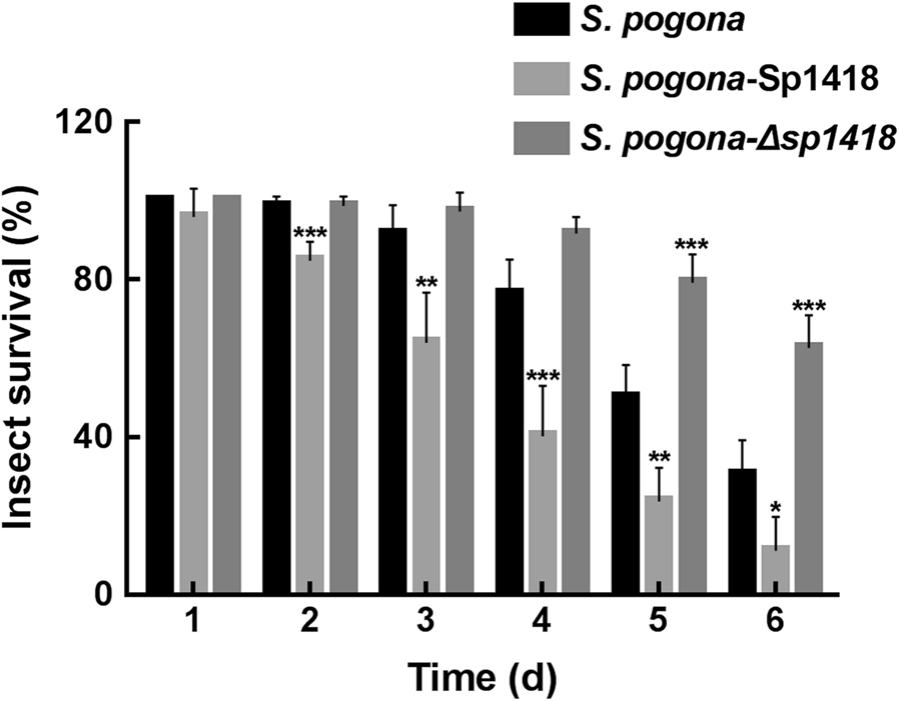
The insecticidal activity against *H. armigera.* After 2 days, the survival rate of *H. armigera* showed a significant decrease, which represents that the fermentation of *S. pogona*-Sp1418 exhibited a stronger toxin to *H. armigera* than that of the wild-type strain, while *S. pogona*-Δ*sp1418* showed weak insecticidal activity. Error bars indicated standard errors of results from n = 3 replicates

To confirm the difference in butenyl-spinosyn productions, we determined the viability of *H. armigera* in feeds mixed with fermentation supernatants of different strains. From the second day, the mortality of *H. armigera* treated with *S. pogona*-Sp1418 fermentation supernatant was significantly higher than that treated with *S. pogona* fermentation supernatant (Fig. 6), and the lethal time (LT_50_) was also advanced by 0.91 days (Additional file 3: Table S2), which showed a significant increase in insecticidal activity. The treatment with *S. pogona*-Δ*sp1418* had a contrasting effect, and the *sp1418* deletion in *S. pogona* resulted in a significant reduction in its insecticidal activity. The result indicated that the high concentration of butenyl-spinosyn had a better contact effect on *H. armigera*, while *S. pogona*-Δ*sp1418* that do not produce butenyl-spinosyn was almost helpless against *H. armigera*.

### Identification and verification of distinctive proteins

**Fig. 7:**
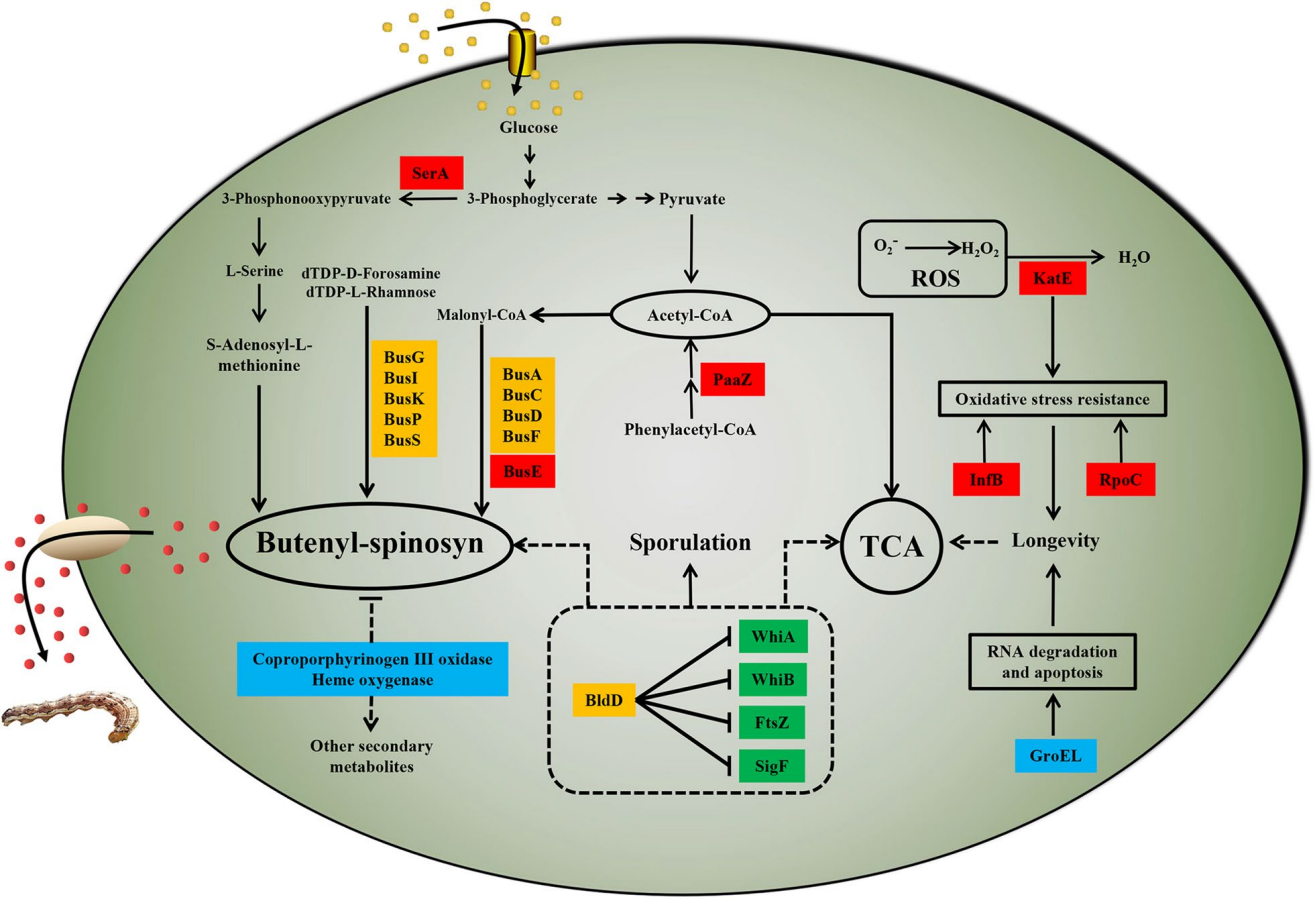
Regulatory network schematic diagram of the sp1418 gene in *S. pogona*. Blue rectangle: downregulated protein in *S. pogona*-Sp1418, red rectangle: upregulated protein in *S. pogona*-Sp1418; green rectangle: transcriptionally downregulated genes in *S. pogona*-Sp1418, yellow rectangle: transcriptionally upregulated genes in *S. pogona*-Sp1418. The upregulation of the *sp1418* gene affected KatE, RpoC, and InfB, which regulate the redox balance and maintain cell growth, avoiding the effects of oxidative stress on cells. The downregulation of the groEL gene could reduce RNA degradation and maintain RNA stability. SerA converts 3-phosphoglycerate to 3-phosphonooxypyruvate, and PaaZ promotes the synthesis of acetyl-CoA, the upregulation of which could generate more precursors for butenyl-spinosyn biosynthesis, and the upregulation of bus genes promoted biosynthesis. Coproporphyrinogen III oxidase and haem oxygenase are related to secondary metabolism, the downregulation of which could provide additional raw materials for butenyl-spinosyn biosynthesis. The expression change in genes involved in sporulation revealed differences in phenotypes and is also associated with primary and secondary metabolism

To analyse the discrepancy in protein levels between different strains, distinctive bands were screened by SDS-PAGE gel (Additional file 1: Figure S4) and identified by 1D-LC–MS/MS. The identified proteins, including catalase (KatE), oxepin-CoA hydrolase (PaaZ) and other proteins, were categorized using UniProt (https://www.uniprot.org) for functional analyses (Table 1). Through KEGG (https://www.kegg.jp) analysis, we determined the metabolic processes involved in these identified proteins (Fig. 7), and three more highly expressed proteins, InfB, RpoC and KatE were found, which were associated with oxidative stress [30–32].

**Table 1:** Proteins identified from SDS-PAGE gel analysis

Protein bands	UniProtKB	Protein description	Gene	MW (KDa)	Possible function
a	A0A0X3SRR8	Polyketide synthase BusE, partial	*busE*	219.9	Polyketide biosynthesis of butenyl-spinosyn
b	A0A2N3XQH8	DNA-directed RNA polymerase subunit beta’	*rpoC*	144.76	DNA-dependent RNA polymerase catalyzes the transcription of DNA into RNA using the four ribonucleoside triphosphates as substrates
c	A0A2N3Y983	Translation initiation factor IF-2	*infB*	105.31	One of the essential components for the initiation of protein synthesis. Protects formylmethionyl-tRNA from spontaneous hydrolysis and promotes its binding to the 30S ribosomal subunits. Also involved in the hydrolysis of GTP during the formation of the 70S ribosomal complex
d	A0A2N3Y379	Oxepin-CoA hydrolase/3-oxo-5,6-dehydrosuberyl-CoA semialdehyde dehydrogenase	*paaZ*	72.19	Oxidoreductase activity, acting on the aldehyde or oxo group of donors, NAD or NADP as acceptor
e	A0A2N3XV92	Catalase	*katE*	55.25	Hydrogen peroxide catabolic process, response to oxidative stress
f	A0A2N3Y9R3	D-3-phosphoglycerate dehydrogenase	*serA*	54.93	This protein is involved in step 1 of the subpathway that synthesizes l-serine from 3-phospho-D-glycerate
g	A0A1H3I9Q3	Molecular chaperone GroEL	*groEL*	59.12	Prevents misfolding and promotes the refolding and proper assembly of unfolded polypeptides generated under stress conditions
h	D9WS96	Coproporphyrinogen III oxidase		43.04	Catalytic activity, iron-sulfur cluster binding
i	A0A0K9XIH0	Heme oxygenase		27.06	Heme oxygenase (decyclizing) activity, metal ion binding

### Determination of the Catalase (CAT) activity and H_2_O_2_ concentration

Based on the analysis of growth and protein expression differences, we speculate that the significant difference of growth between the engineered strains and the wild-type strain is closely related to the changes in the CAT activity and H_2_O_2_ content. By detecting the CAT activity and the concentration of hydrogen peroxide (Table 2), the ability of hydrogen peroxide metabolism in the wild-type and engineered strains were identified. As expected, the CAT activity of the knockout strain was obviously lower than that of the wild-type bacteria, and the content of H_2_O_2_ was 1.67 times that of the wild-type and 4.51 times that of the overexpression strain. While the overexpression strain was just the opposite, its CAT activity was 1.24 times that of the wild-type and 3.16 times that of the knockout strain, which was in line with the expectation of SDS-PAGE analysis.

**Table 2:** CAT activity and H_2_O_2_ concentration of *S. pogona*, *S. pogona*-Δ*sp1418* and *S. pogona*-Sp1418

Strains	CAT activity (U)	H_2_O_2_ concentration (μmol/g)
*S. pogona*	204.95 ± 14.23	1.149 ± 0.131
*S. pogona*-Δ*sp1418*	80.29 ± 23.14**	1.916 ± 0.304**
*S. pogona*-Sp1418	253.86 ± 12.95*	0.425 ± 0.062**

The CAT activity (U) is expressed as the change in absorbance per minute per gram of bacteria

* and ** indicated P < 0.05 and P < 0.005, respectively, compared to *S. pogona* under the same conditions

## Discussion

The structure and biosynthesis of spinosyn analogues have been clearly described [4, 33], and studies on the transformation of its gene clusters and the improvement of heterologous expression have been studied [7, 34, 35]; however, the metabolic situation in vivo is complex, and any slight metabolic disturbance will lead to complex changes, which may produce unintended consequences [11]. Therefore, the analysis of the genome characteristics of *S. pogona* and related regulatory proteins may help to understand their gene expression preferences. The global regulatory network is of great significance for the use of genetic modification to increase the production of butenyl-spinosyn and to reduce the negative effects of metabolic disturbances.

There are many studies on TetR family proteins, which have been found to play an important role in the regulation of secondary metabolite biosynthesis, transporter regulation, and regulation of related operators [36–38]. There are dozens of tetR family proteins in the genome of *S. pogona*, and their protein sizes, amino acid sequences and active sites are also different, which determines their functional diversity.

Here, we identified and characterized a transcriptional regulator in the growth and butenyl-spinosyn biosynthesis of *S. pogona*. Using the two-way verification of knockout and overexpression, we determined the potential mode of action of Sp1418. The sporulation ability observation, growth curve detection and HPLC results showed that Sp1418 played a regulatory role in cell growth, differentiation and secondary metabolism in *S. pogona*.

By distinctive protein analysis, we determined that Sp1418 had an effect on CAT, RpoC and InfB, the three proteins are closely related to the regulation of reactive oxygen species (ROS) and oxidative stress in vivo, which cause protein structure mutations or the loss of biological activity, DNA strand breaks, DNA site mutations, DNA double-strand aberrations, etc., eventually leading to oxidative injury [39]. However, studies have indicated that DNA injury can also induce ROS production, and there is a close relationship between the processes [40]. We speculate that the restricted growth of *S. pogona*-Δ*sp1418* is most likely due to the DNA injury and improved ROS level. The CAT activity and H_2_O_2_ content were detected in the mutants and the wild-type strains, revealing that *S. pogona*-Sp1418 had increased CAT activity and a reduced H_2_O_2_ concentration. CAT activity affects oxidative stress, which can disrupt the intracellular redox balance, thereby activating or inhibiting many signalling pathways and some signal-mediated molecules. Nrf2/Keap1 participates in the important signalling pathway for intracellular resistance to oxidative stress and maintaining redox balance [32, 41]. In the genome, there were genes in the vicinity of *sp1418* that were predicted to express nuclear transport factor 2 family proteins. In addition, in the qRT-PCR experiment, we found that the expression of *sigF* was most significantly increased in *S. pogona*-Sp1418, and this gene was confirmed to be associated with H_2_O_2_ tolerance in the previous study [27]. Hence, we can reasonably speculate that the abnormal expression of Sp1418 disrupts the redox balance in cells, resulting in differences in the expression of related proteins, which had an effect on growth, sporulation and secondary metabolite biosynthesis.

SerA converts 3-phosphoglycerate to 3-phosphonooxypyruvate, and ultimately promotes the synthesis of S-adenosyl-l-methionine, which serves as a coenzyme of ubiquitous methyltransferases and plays an essential role in butenyl-spinosyn production [42]. The *bus* families are key genes responsible for the biosynthesis of butenyl-spinosyn, and these genes’ expression level greatly affects the synthesis efficiency of butenyl-spinosyn. The qRT-PCR analysis showed that the transcription level of most *bus* genes was significantly increased in overexpression strain, and *S. pogona*-Sp1418 was exhibited a notably higher abundance of BusE than other strains by SDS-PAGE analysis (Table 1, Additional file 1: Figure S3). The molecular chaperone GroEL is involved in RNA degradation, and the downregulation of *groEL* expression levels in *S. pogona*-Sp1418 helps maintain RNA stability, which may be responsible for the high transcription levels of the *bus* genes. Also, we identified the PaaZ protein in the differential bands and found that its expression promotes the synthesis of acetyl-CoA by KEGG analysis, which is associated with the metabolism of butenyl-spinosyn. Therefore, the butenyl-spinosyn production was significantly increased in *S. pogona*-Sp1418 (Fig. 7).

## Conclusion

In summary, Sp1418 is an important global regulator in *S. pogona* that affects the sporulation, growth, and butenyl-spinosyn biosynthesis, which was likely due to the disturbed redox balance and abnormally expressed enzymes. During the fermentative process, oxidative stress is the most serious threat to the synthesis of cell survival [43], which has a prominent impact on the physiological and biochemical changes. In our study, the proteins CAT, RpoC and InfB involved in oxidative stress are speculated to be the main factor causing the alteration of the engineering strains, and their expression changes were regulated by Sp1418. The result makes contributions to understand the function of tetR transcriptional regulatory factors. When industrial production was carried out, we could consider improving the antioxidant and DNA repair capacity of microorganisms, which was important for maintaining cell growth and stable metabolite expression.

## Materials and methods

### Bacterial strains, plasmids, media, growth conditions

The bacterial strains, plasmids, and primers used in this study are listed in Additional file 4: Tables S3 and S4. The spores of *S. pogona* NRRL 30,141 were cultivated in activation medium (per liter: 10 g glucose; 45 g trypticase soy broth; 9 g yeast extract; 2.2 g MgSO_4_), with a starting volume of 50 mL (30 °C, 200 rpm). After cultivating of the strains for 48 h, 2.5 mL of a bacterial suspension was added to 50 mL of fermentation medium (per liter: 1 g KNO_3_; 0.01 g FeSO_4_; 0.5 g K_2_HPO_4_; 0.5 g MgSO_4_; 20 g glucose; 4 g yeast extract; 4 g tryptone; pH 7.2) and cultured at 30 °C with 200 rpm. The culture conditions of mutants were the same as those for the wild strains with antibiotics in the medium (apramycin, 50 mg/L). All *Escherichia coli* (*E. coli*) strains were grown in lysogeny broth (LB) at 37 °C supplemented with antibiotics as required (apramycin, 50 mg/L).

### Construction and verification of the recombinant strains

To produce the pKCcas9d-sgRNA-UHA-DHA, primer pair sgRNA-F/sgRNA-R was designed (Sangon, Shanghai, China) to amplify the sgRNA from pKCcas9dO, and primer pairs tet-up-F/tet-up-R and tet-down-F/tet-down-R were designed to amplify the upstream and downstream sequences of the *sp1418* gene from *S. pogona* genomic DNA as homologous arms. Then, the PCR fragments were fused by overlap extension PCR using primers sgRNA-F/tet-down-R. The *Spe*I and *Hin*dIII cut fusion fragment was cloned in pKCcas9dO plasmid digested with same enzymes.

The *P*_*ermE*_ gene was amplified from plasmid pOJ260-*cm*-*P*_*ermE*_ by usng primers perm-F/perm-R, and *sp1418* gene amplified by primer pair tetR-F/tetR-R from genomic DNA of *S. pogona.* The amplified products were fused by overlap extension PCR by usng primers perm-F/tetR-R, and the fusion fragment was cloned into the corresponding restriction sites of pOJ260 after enzyme digestion (*Xba*I and *Hin*dIII), yielding recombinant plasmid pOJ260-*P*_*ermE*_-*sp1418* (Additional file 1: Figure S5).

These cloned plasmids were transferred into *S. pogona* by standard conjugation methods [44] and yielded recombinant strains *S. pogona*-Δ*sp1418* and *S. pogona*-Sp1418 (Additional file 1: Figures S6, S7).

### Cultivation profile analysis of the wild-type and recombinant strains

To monitor the growth profiles and phenotypic differences, growth curve determination and morphological observations of the wild-type and recombinant strains were performed [9]. During the fermentation, the butenyl-spinosyn was detected by HPLC every day [45]. 500 μL of fermentation broth was mixed with ethyl acetate. After 1 h of extraction at 60 °C, the supernatant was lyophilized and added 50 μL methanol, which was centrifuged at 10,000 rpm for 5 min, and the supernatant was identified by HPLC. A 20 μL aliquot of each supernatant was loaded onto a C18 column (AQ12S05-1546WT) and eluted with the elution buffer at 1.0 mL/min. The elution buffer A contained 10% (v/v) acetonitrile, and elution buffer B contained 90% (v/v) acetonitrile. The detection wavelength was set at 250 nm during the analysis. To detect the insecticidal activity of butenyl-spinosyn against *H. armigera,* 1 mL fermentation supernatant of different strains was mixed with 19 mL feed separately (per liter: 40 g yeast extract; 70 g bean flour; 5 g vitamin C; 15 g agar; 1 g sorbic acid; and 10 g penicillin) and evenly distributed in 24-well plates [9], and the survival percentage was recorded.

### Protein extraction and SDS-PAGE analysis

To extract total protein from whole cells, which were harvested at different time points for the wild-type and recombinant strains. After measuring the concentration of the protein by Bradford assay, the profiles of the protein samples were checked by SDS-PAGE.

### Heterologous expression and Western blot analysis of the Sp1418 protein

To verify the expression of Sp1418 in the wild-type and recombinant strains, the *sp1418* gene fragment was amplified by using primers tetR-H-F/tetR-H-R and cloned into the pET28a vector, and transferred to *E. coli* BL21. Heterologously expressed protein was harvested from the recombinant strain cultured in LB supplemented with 40 μg/mL kanamycin. The anti-Sp1418 antibody was obtained by immunizing rabbits and the *sp1418* expression level in the wild-type and recombinant strains was analysed via Western blot [46].

### Nano-LC–MS/MS analysis

Differential protein bands of whole cell protein analysis and heterologously expressed protein Sp1418 were excised from the SDS-PAGE gel for in-gel tryptic digestion and subsequently liquid chromatography-tandem mass spectrometry (LC–MS/MS) analysis [44]. The 1D-LC–MS/MS analysis was performed by using an LTQ XL mass spectrometer (Thermo Fisher, San Jose, CA, USA).

### CAT activity and H_2_O_2_ concentration detection

After 4 days, cells were collected from the wild-type and recombinant strains. To test the CAT activity, 0.1 g cells were taken after centrifugation, which were grind with liquid nitrogen and resuspended in PBS (pH 7.0). After centrifugation at 6000 r/min for 10 min, 450 μL supernatant was taken, added to 1.5 mL PBS (pH 7.8) containing 1% (m/v) polyvinylpyrrolidone and 1.0 mL deionized water, after warming up at 25 °C, 50 μL 0.1 mol/L H_2_O_2_ solution was added. The absorbance change in the solution was recorded every 0.5 min, and the detection wavelength was 240 nm. To detect the H_2_O_2_ concentration, 0.1 g cells were dissolved in 1.0 mL cold acetone and ultrasonication, the supernatant was collected after centrifugation (8000*g*, 10 min, 4 °C) and performed following the H_2_O_2_ concentration kit instructions (Solarbio, China). The reaction principle is that H_2_O_2_ reacts with titanium sulfate to form a yellow titanium peroxide complex, which has a characteristic absorption at 415 nm.

### Total RNA isolation and qRT-PCR analysis

For transcriptional analysis experiments, the total RNA from the wild-type and recombinant strains from different time points (48, 96 and 144 h) was separately collected following instructions by using a TotalRNAExtractor (Sangon, Shanghai, China). RNA concentration and purity were determined by a NanoDrop 2000 spectrophotometer (Thermo Fisher Scientific, Waltham, MA, USA). DNase treatment and cDNA synthesis were performed by the PrimeScript™ RT Reagent Kit with gDNA Eraser (Takara, Kyoto, Japan) according to the manufacturer’s instructions. Real time qPCR amplification was performed by using SYBR^®^ Permix Ex Tag™ GC (Takara, Kyoto, Japan), and the transcriptional level was assayed on 7500 Real-Time PCR system instruments (Applied Biosystems, USA). The 16S rRNA gene was employed as an internal control to quantify the relative expression of target genes.

### Statistical analysis

SPSS statistics version 19.0 was used to carry out all statistical analyses. A probability value of P < 0.05 was considered statistically significant.

## Supplementary information

**Additional file 1: Figure S1.** Mass spectrum identification of butenyl-spinosyns. MS identification results showed that MS parent ion [M+H]^+^ = 633 (black arrow) contained 617 (M+H^+^) (m/z) ion data and a rhamnose ion fragment of 189 molecular mass, which was confirmed as a butenyl-spinosyn component. **Figure S2.** The sporulation phenotypes in wild type, *S. pogona*-Δ*sp1418* and *S. pogona*-Sp1418. *S. pogona*-Sp1418 did not produce spores or was almost invisible, and the amount of spores of *S. pogona*-Δ*sp1418* is much more than that of *S. pogona*, but there was no significant difference in the spore morphology of *S. pogona* and *S. pogona*-Δ*sp1418*. **Figure S3.** Tricine-SDS-PAGE analysis of heterologously expressed protein Sp1418. Coomassie Brilliant Blue staining of Tricine**-**SDS-PAGE showing heterologous protein Sp1418 expressed in the supernatants of *E. coli* BL21 bearing recombinant plasmid after IPTG induction and ultrasonication. M: 66 kDa protein marker; 1: Samples from *E. coli* BL21 as a negative control 2: Samples from *E. coli* BL21 contained the recombinant plasmid. **Figure S4.** SDS-PAGE gel analysis of total proteins. M: Protein marker; 1: Samples from 96 h *S. pogona*; 2: Samples from 96 h *S. pogona*-Sp1418 cells; 3: Samples from 96 h *S. pogona*-Δ*sp1418* cells. Compared with the three strains, there are many distinctive bands, and totally 9 proteins were identified via 1D-LC–MS/MS. **Figure S5.** Construction of pKCcas9d-sgRNA-UHA-DHA and pOJ260-*P*_*ermE*_-*sp1418*. A. Construction of plasmid pKCcas9d-sgRNA-UHA-DHA; B. Construction of plasmid pOJ260-*P*_*ermE*_-*sp1418.***Figure S6.** Recombination schematic diagram of vetor pOJ260-*P*_*ermE*_-*sp1418* and pKCcas9d-sgRNA-UHA-DHA. A. Recombination schematic diagram of vetor pOJ260-*P*_*ermE*_-*sp1418*; B. Recombination schematic diagram of vetor pKCcas9d-sgRNA-UHA-DHA. **Figure S7.** Identification of *S. pogona*-Δ*sp1418* and *S. pogona*-Sp1418. A: Identification of *P*_*ermE*_-*sp1418* fragment in *S. pogona and S. pogona*-Sp1418. M: DL 2000 DNA marker; 1: PCR products of *S. pogona* with primer pair perm-F/tetR-R; 2: PCR products of *S. pogona*-Sp1418 with primer pair perm-F/tetR-R; B: PCR amplification of *aac(3)*IV gene in *S. pogona and S. pogona*-Sp1418. M: DL 2000 DNA marker; 1–2: PCR products of *S. pogona* with primer pair Apr-F/Apr-R; 3: PCR products of *S. pogona*-Sp1418 with primer pair Apr-F/Apr-R; C: Identification of *aac(3)*IV gene in *S. pogona and S. pogona*-Δ*sp1418*. M: DL 2000 DNA marker; 1: PCR products of *S. pogona* with primers Apr-F/Apr-R; 2: PCR products of *S. pogona*-Δ*sp1418* with primers Apr-F/Apr-R; D: Identification of *sp1418* gene in *S. pogona and S. pogona*-Δ*sp1418* M: DL 2000 DNA marker; 1: PCR products of *S. pogona* with primers tetR-P-F/tetR-P-R; 2: PCR products of *S. pogona*-Δ*sp1418* with primers tetR-P-F/tetR-P-R.

**Additional file 2: Table S1.** Heterologously expressed protein Sp1418 identified by 1D-LC–MS/MS.

**Additional file 3: Table S2.** Biological insecticidal activity of *S. pogona*, *S. pogona*-Δ*sp1418* and *S. pogona*-Sp1418.

**Additional file 4: Table S3.** Primers, plasmids and strains used in this study. **Table S4.** qRT-PCR primers used in this study.

## Data Availability

All data generated or analysed during this study are included in this published article and its additional files.

## Abbreviations

ROS: Reactive oxygen species
CAT: Catalase
UHA: Upstream homology arm
DHA: Downstream homologous arm
LC–MS/MS: Liquid chromatography–tandem mass spectrometry
Pnp: Polynucleotide phosphorylase
