# Supplementary material for: Effect of the TetR family transcriptional regulator Sp1418 on the global metabolic network of Saccharopolyspora pogona
Source: Microb Cell Fact. 2020 Feb 11;19:27. doi: 10.1186/s12934-020-01299-z (PMC7011500; doi:10.1186/s12934-020-01299-z)
Supplement: Supplementary file 1 — Additional file 1: Figure S1. Mass spectrum identification of butenyl-spinosyns. MS identification results showed that MS parent ion [M+H]+ = 633 (black arrow) contained 617 (M+H+) (m/z) ion data and a rhamnose ion fragment of 189 molecular mass, which was confirmed as a butenyl-spinosyn component. Figure S2. The sporulation phenotypes in wild type, S. pogona-Δsp1418 and S. pogona-Sp1418. S. pogona-Sp1418 did not produce spores or was almost invisible, and the amount of spores of S. pogona-Δsp1418 is much more than that of S. pogona, but there was no significant difference in the spore morphology of S. pogona and S. pogona-Δsp1418. Figure S3. Tricine-SDS-PAGE analysis of heterologously expressed protein Sp1418. Coomassie Brilliant Blue staining of Tricine-SDS-PAGE showing heterologous protein Sp1418 expressed in the supernatants of E. coli BL21 bearing recombinant plasmid after IPTG induction and ultrasonication. M: 66 kDa protein marker; 1: Samples from E. coli BL21 as a negative control 2: Samples from E. coli BL21 contained the recombinant plasmid. Figure S4. SDS-PAGE gel analysis of total proteins. M: Protein marker; 1: Samples from 96 h S. pogona; 2: Samples from 96 h S. pogona-Sp1418 cells; 3: Samples from 96 h S. pogona-Δsp1418 cells. Compared with the three strains, there are many distinctive bands, and totally 9 proteins were identified via 1D-LC–MS/MS. Figure S5. Construction of pKCcas9d-sgRNA-UHA-DHA and pOJ260-PermE-sp1418. A. Construction of plasmid pKCcas9d-sgRNA-UHA-DHA; B. Construction of plasmid pOJ260-PermE-sp1418.Figure S6. Recombination schematic diagram of vetor pOJ260-PermE-sp1418 and pKCcas9d-sgRNA-UHA-DHA. A. Recombination schematic diagram of vetor pOJ260-PermE-sp1418; B. Recombination schematic diagram of vetor pKCcas9d-sgRNA-UHA-DHA. Figure S7. Identification of S. pogona-Δsp1418 and S. pogona-Sp1418. A: Identification of PermE-sp1418 fragment in S. pogona and S. pogona-Sp1418. M: DL 2000 DNA marker; 1: PCR products of S. pogona with prime [file 12934_2020_1299_MOESM1_ESM.doc]

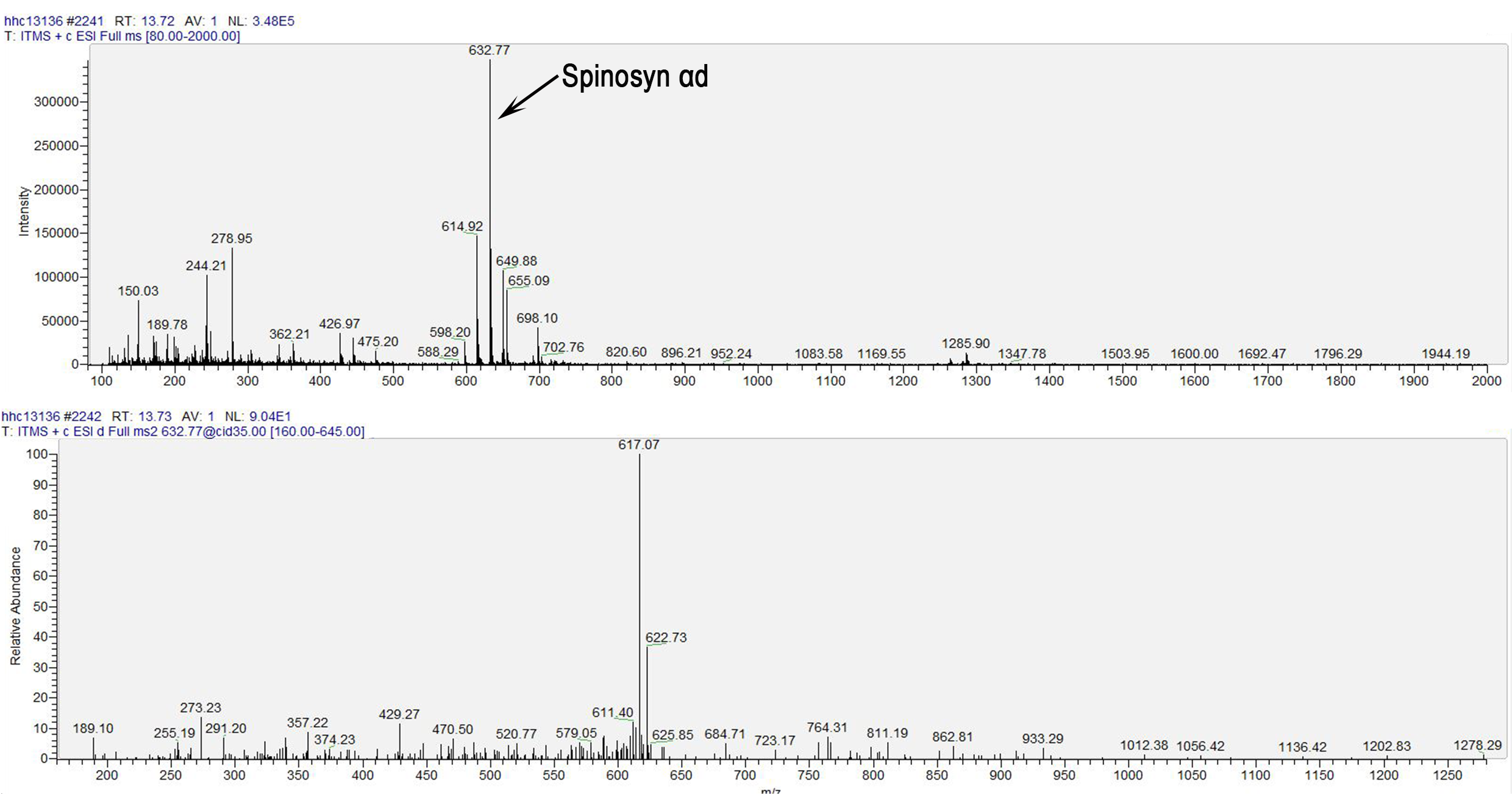
**Figure S1. Mass spectrum identification of butenyl-spinosyns.** MS identification results showed that MS parent ion [M+H]^+^ = 633 (black arrow) contained 617 (M+H^+^) (m/z) ion data and a rhamnose ion fragment of 189.1 molecular mass, which was confirmed as a butenyl-spinosyn component.


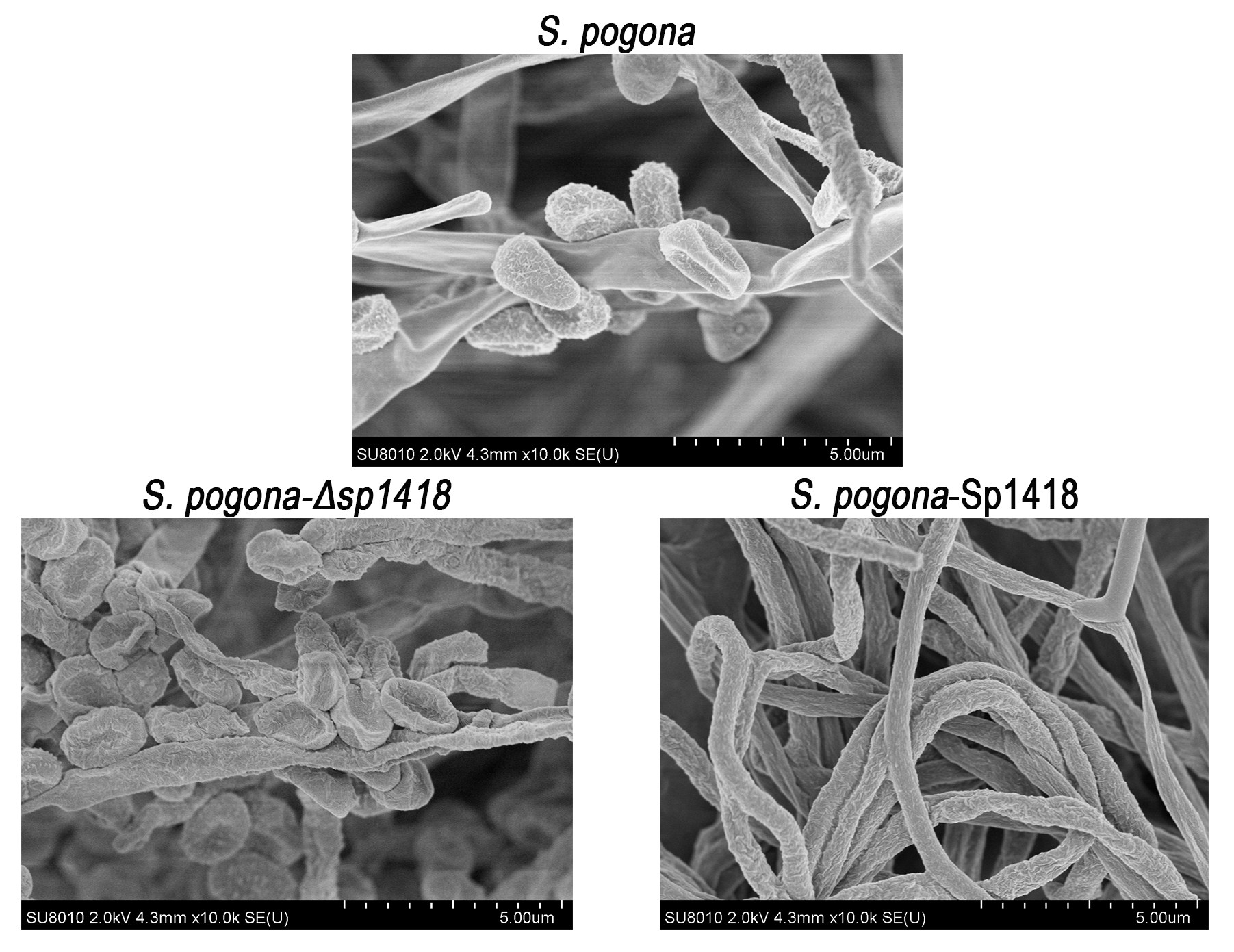


**Figure S2. The sporulation phenotypes in wild type, *S. pogona-*Δ*sp1418* and *S. pogona*-Sp1418.** *S. pogona*-Sp1418 did not produce spores or was almost invisible, and the amount of spores of *S. pogona-*Δ*sp1418* was much more than that of *S. pogona*, but there was no significant difference in the spore morphology of *S. pogona* and *S. pogona-*Δ*sp1418*.


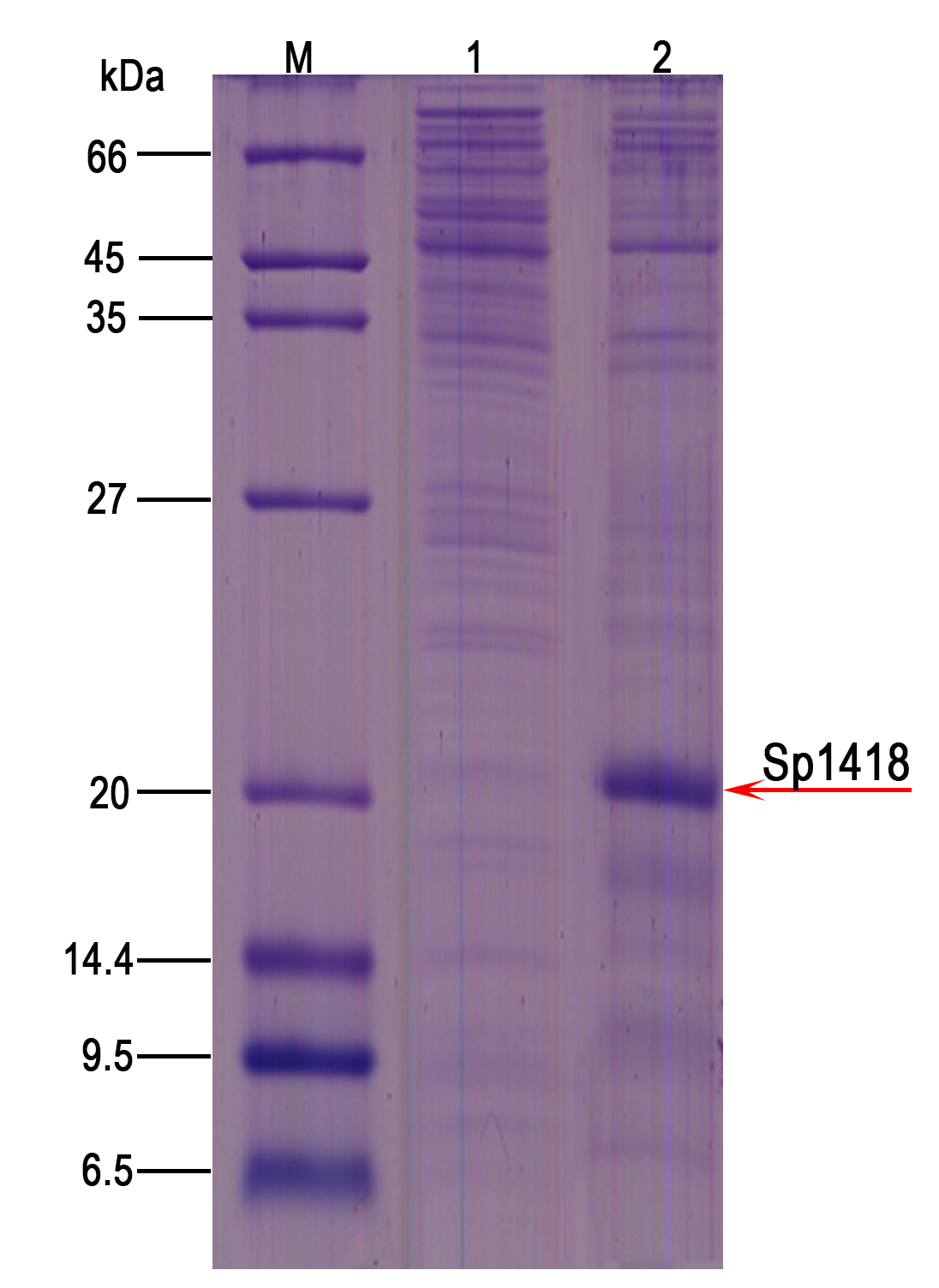
**Figure S3.** **Tricine**-**SDS-PAGE analysis of heterologously expressed protein Sp1418.** Coomassie Brilliant Blue staining of Tricine**-**SDS-PAGE showing heterologous protein Sp1418 expressed in the supernatants of *E. coli* BL21 bearing recombinant plasmid after IPTG induction and ultrasonication.

M: 66 kDa protein marker;

1: Samples from *E. coli* BL21 as a negative control

2: Samples from *E. coli* BL21 contained the recombinant plasmid.


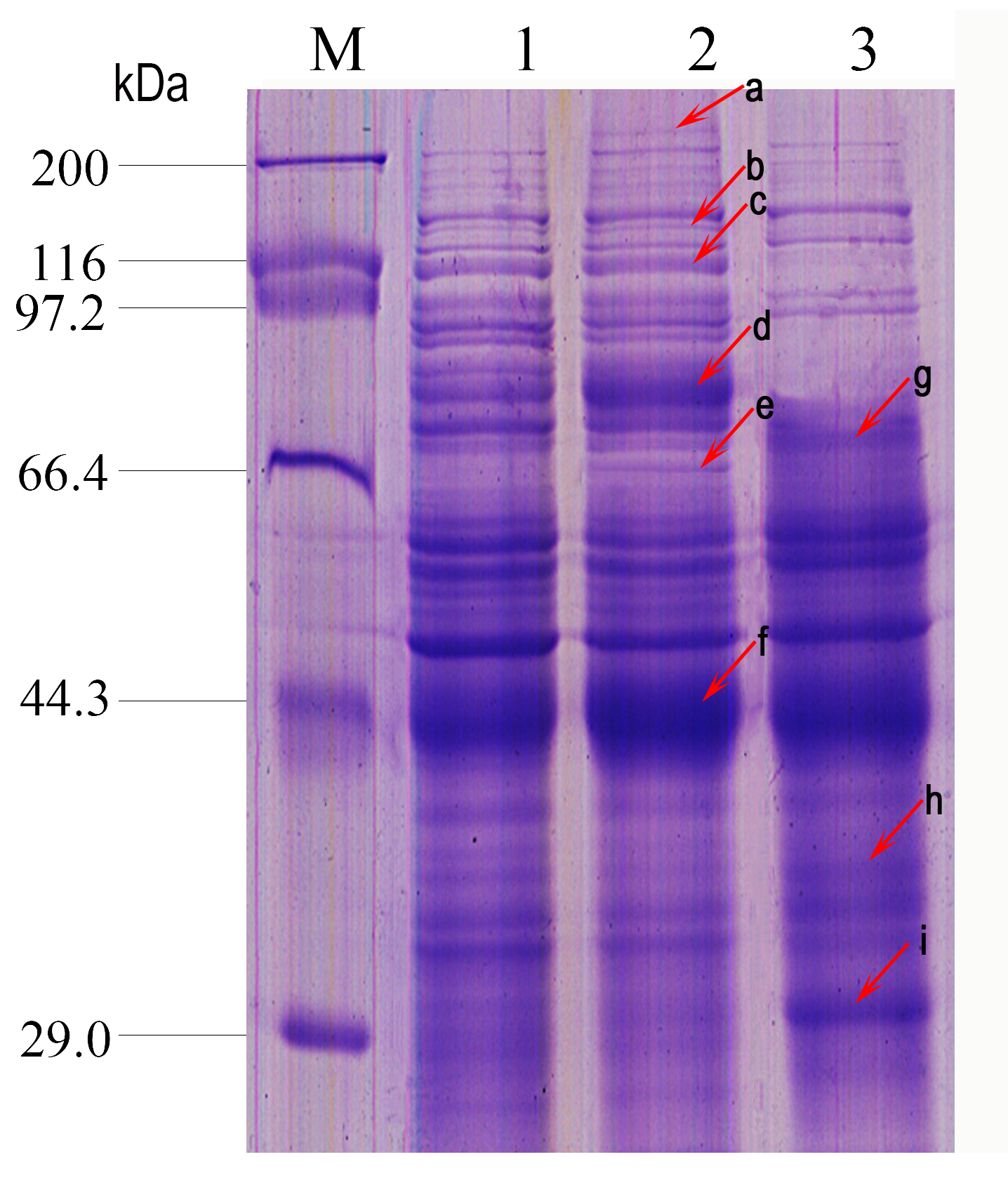
**Figure S4. SDS-PAGE gel analysis of total proteins.**

M: Protein marker;

1: Samples from 96 h *S. pogona*;

2: Samples from 96 h *S. pogona*-Sp1418 cells;

3: Samples from 96 h *S. pogona-*Δ*sp1418* cells.

Compared with the three strains, there are many distinctive bands, and totally 9 proteins were identified via 1D-LC-MS/MS.


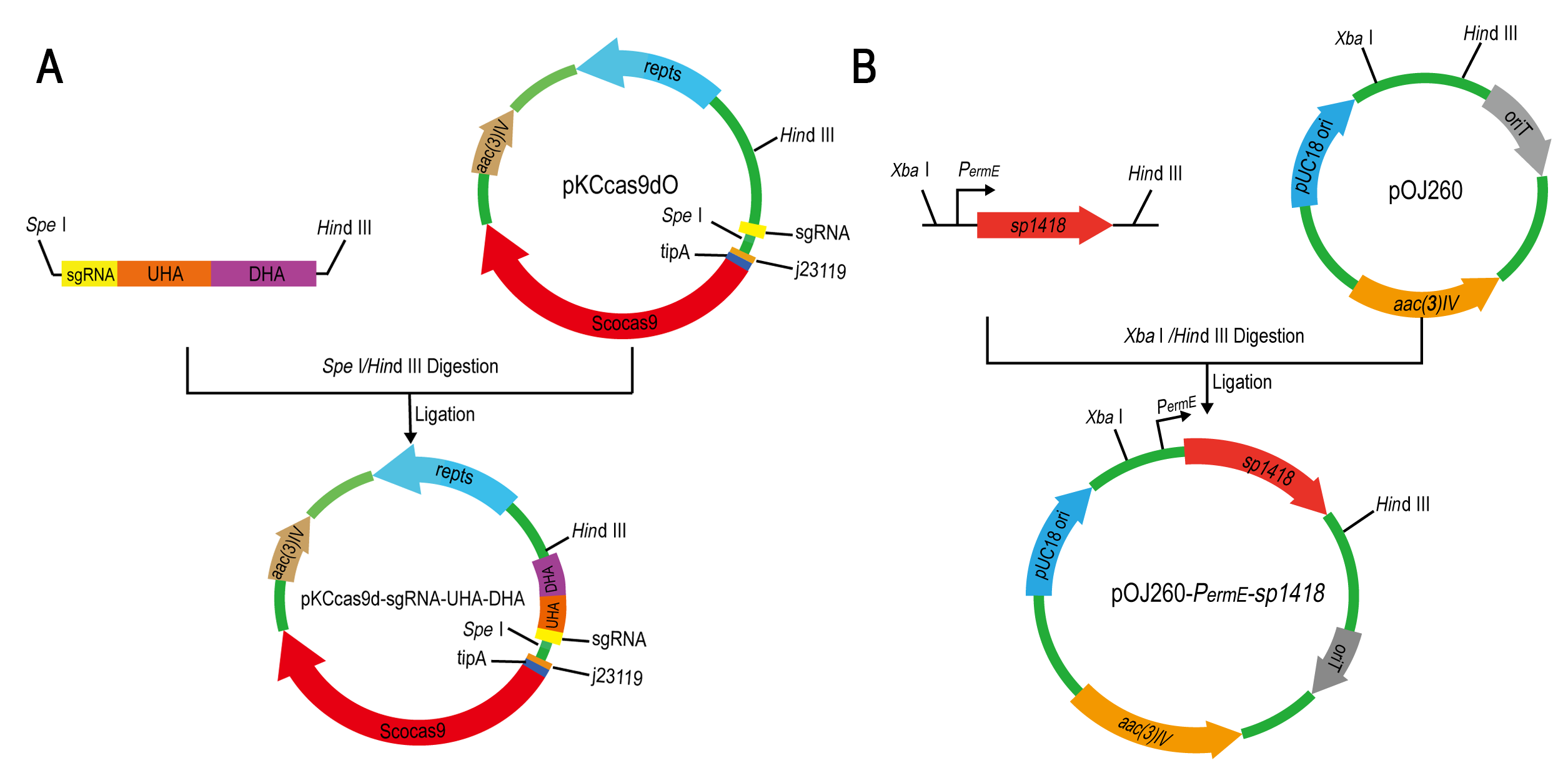
**Figure S5. Construction of pOJ260-*P_ermE_-sp1418* and pKCcas9d-sgRNA-UHA-DHA.**

A. Construction of plasmid pKCcas9d-sgRNA-UHA-DHA

B. Construction of plasmid pOJ260-*P_ermE_-sp1418*


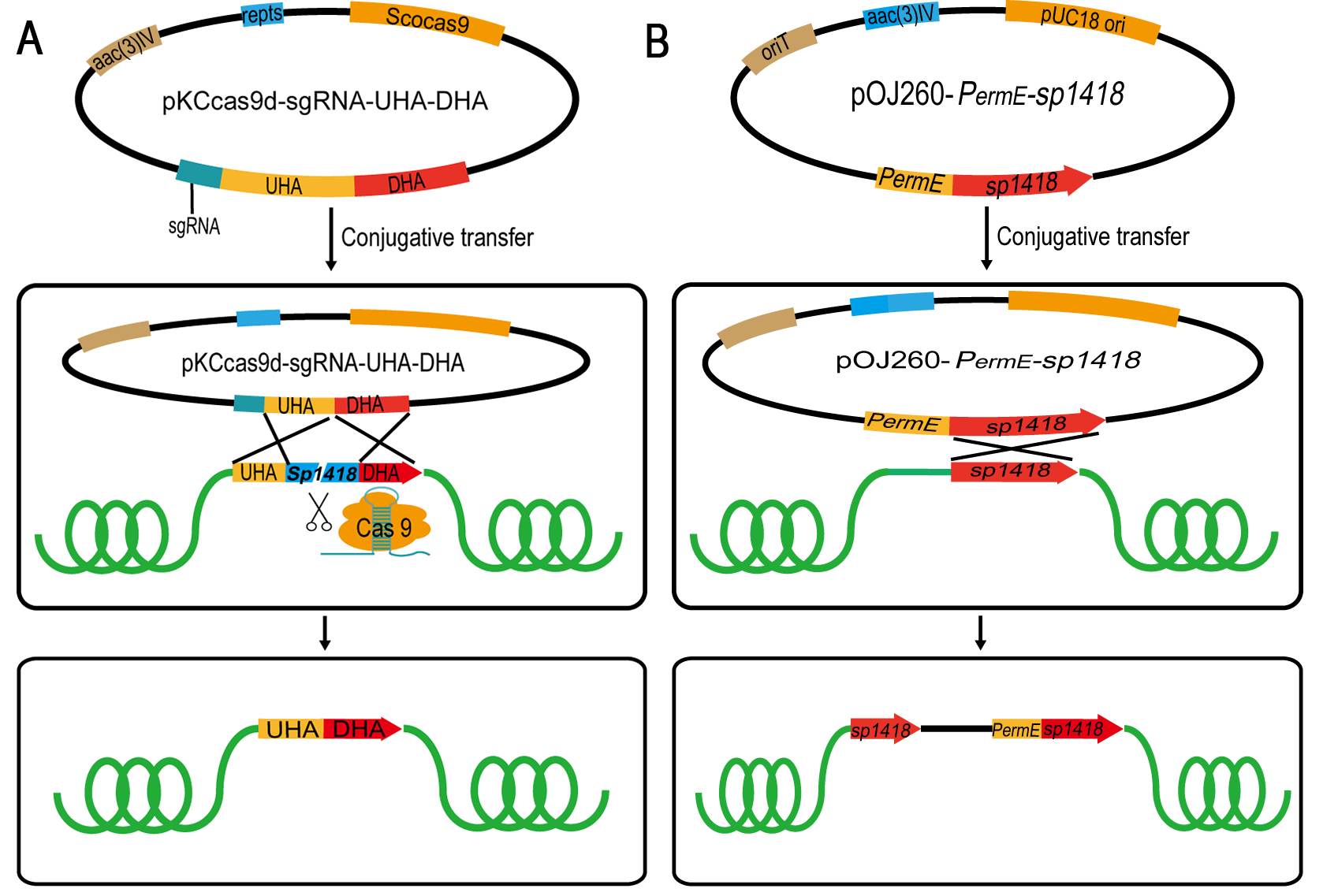
**Figure S6. Recombination schematic diagram of vetor pOJ260-*P_ermE_-sp1418* and pKCcas9d-sgRNA-UHA-DHA*.***

A. Recombination schematic diagram of vetor pOJ260-*P_ermE_-sp1418*;

B. Recombination schematic diagram of vetor pKCcas9d-sgRNA-UHA-DHA.


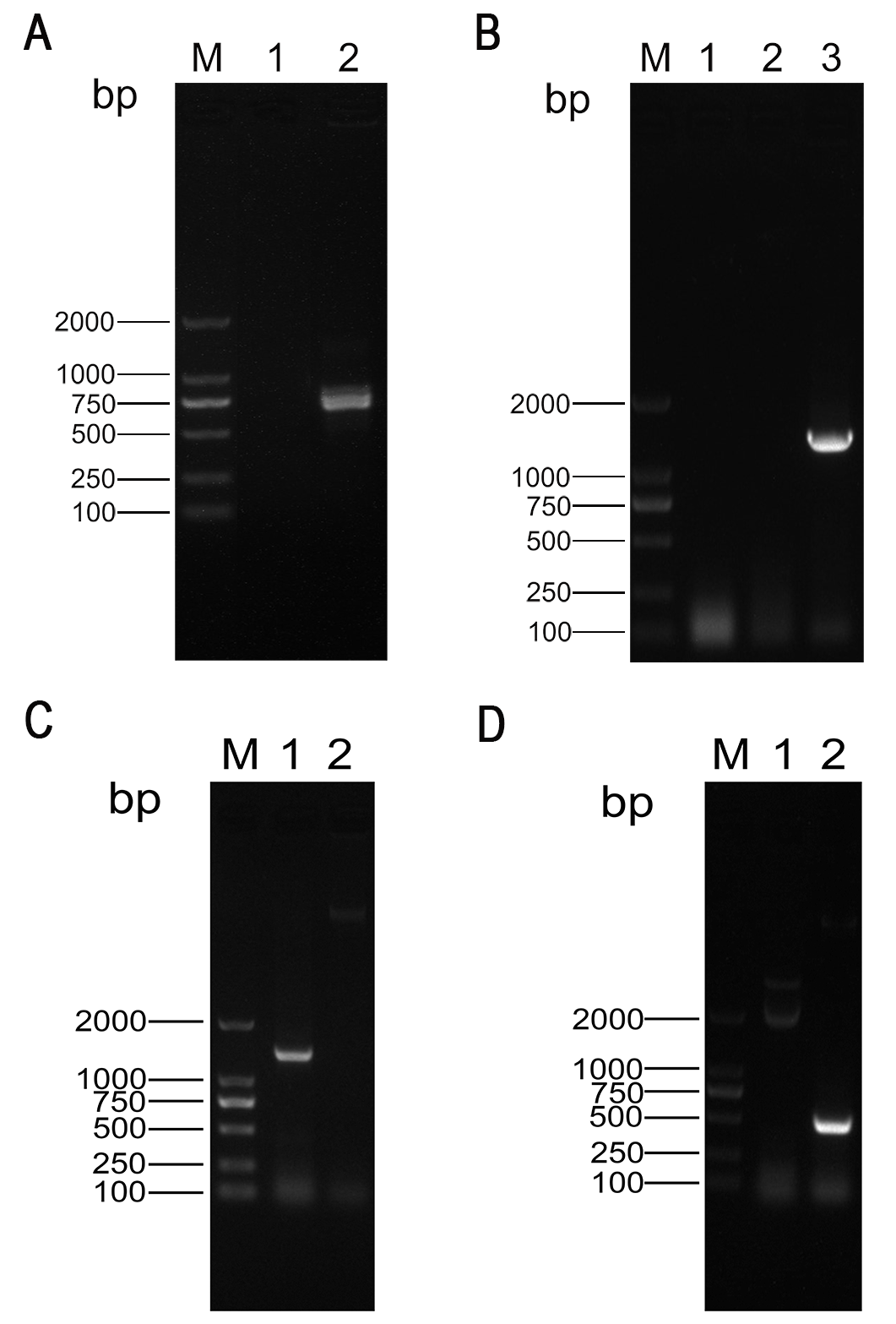
**Figure S7.** **Identification of** ***S. pogona-*Δ*sp1418* and *S. pogona*-Sp1418.**

A: Identification of *P_ermE_-sp1418* fragment in *S. pogona and S. pogona*-Sp1418.

M: DL 2000 DNA marker;

1: PCR products of *S. pogona* with primer pair perm-F/tetR-R;

2: PCR products of *S. pogona*-Sp1418 with primer pair perm-F/tetR-R;

B: PCR amplification of *aac(3)*IV gene in *S. pogona and S. pogona*-Sp1418.

M: DL 2000 DNA marker;

1-2: PCR products of *S. pogona* with primer pair Apr-F/Apr-R;

3: PCR products of *S. pogona*-Sp1418 with primer pair Apr-F/Apr-R;

C: Identification of *aac(3)*IV gene in *S. pogona and S. pogona-*Δ*sp1418*.

M: DL 2000 DNA marker;

1: PCR products of *S. pogona* with primers Apr-F/Apr-R;

2: PCR products of *S. pogona-*Δ*sp1418* with primers Apr-F/Apr-R;

D: Identification of *sp1418* gene in *S. pogona and S. pogona-*Δ*sp1418*

M: DL 2000 DNA marker;

1: PCR products of *S. pogona* with primers tetR-P-F/tetR-P-R;

2: PCR products of *S. pogona-*Δ*sp1418* with primers tetR-P-F/tetR-P-R.
