# Supplementary material for: Effect of the TetR family transcriptional regulator Sp1418 on the global metabolic network of Saccharopolyspora pogona
Source: Microb Cell Fact. 2020 Feb 11;19:27. doi: 10.1186/s12934-020-01299-z (PMC7011500; doi:10.1186/s12934-020-01299-z)
Supplement: Supplementary file 2 — Additional file 2: Table S1. Heterologously expressed protein Sp1418 identified by 1D-LC–MS/MS. [file 12934_2020_1299_MOESM2_ESM.doc]

| Accession | Description | Score | Coverage | Proteins | Unique Peptides | Peptides | #PSMs | AAs | MW [kDa] | Calc. pI |
| --- | --- | --- | --- | --- | --- | --- | --- | --- | --- | --- |
| WP_010314669.1 | TetR family transcriptional regulator | 194.47 | 34.63 | 1 | 2 | 11 | 118 | 231 | 26.3 | 8.98 |

Table S1. Heterologously expressed protein Sp1418 identified by 1D-LC-MS/MS
