# Supplementary material for: Effect of the TetR family transcriptional regulator Sp1418 on the global metabolic network of Saccharopolyspora pogona
Source: Microb Cell Fact. 2020 Feb 11;19:27. doi: 10.1186/s12934-020-01299-z (PMC7011500; doi:10.1186/s12934-020-01299-z)
Supplement: Supplementary file 3 — Additional file 3: Table S2. Biological insecticidal activity of S. pogona, S. pogona-Δsp1418 and S. pogona-Sp1418. [file 12934_2020_1299_MOESM3_ESM.doc]

Table S2. Biological insecticidal activity of *S. pogona*, *S. pogona-*Δ*sp1418* and *S. pogona*-Sp1418

| **Strains** | **Relative coefficient（R^2^）** | **LT_50_ (d)** | **95% Confidence interval** |
| --- | --- | --- | --- |
| ***S. pogona*** | **0.8665** | **5.081** | **4.838-5.367** |
| ***S. pogona*-Sp1418** | **0.8549** | **3.759** | **3.512-4.012** |
| ***S. pogona-*Δ*sp1418*** | **0.9785** | **6.607** | **6.103-7.432** |
