# Supplementary material for: Effect of the TetR family transcriptional regulator Sp1418 on the global metabolic network of Saccharopolyspora pogona
Source: Microb Cell Fact. 2020 Feb 11;19:27. doi: 10.1186/s12934-020-01299-z (PMC7011500; doi:10.1186/s12934-020-01299-z)
Supplement: Supplementary file 4 — Additional file 4: Table S3. Primers, plasmids and strains used in this study. Table S4. qRT-PCR primers used in this study. [file 12934_2020_1299_MOESM4_ESM.doc]

|  | **Relative description** | **Sources** |
| --- | --- | --- |
| **Strains** |  |  |
| *E.coli* Top10 | Host for general cloning | Lab store |
| *E.coli* S17-1 | Donor strains for conjugation | Lab store |
| *S.pogona* NRRL 30141 | The producer strains of butenyl-spinosyn | Lab store |
| *S. pogona-*Sp1418 | *S.pogona* harboring pOJ260-*P_ermE_-sp1418* | This work |
| *S. pogona-*Δ*sp1418* | *sp1418* knockout mutant of *S.pogona* | This work |
| **Plasmids** |  |  |
| pOJ260 | *E.coli*-cloning vector, containing pUC18 replicon, oriT, Apra^R^ | Lab store |
| pOJ260-cm- *P_ermE_* | Containing *P_ermE_* sequence | Lab store |
| pOJ260- *P_ermE_* -*sp1418* | *P_ermE_* -*sp1418* inserted into pOJ260 by *Xba*I and *Hin*dIII | This work |
| pKCcas9dO | E. coli-cloning vector, containing Scocas9, oriT, Apr^R^ | Lab store |
| pKCcas9d-sgRNA-UHA-DHA | sgRNA-UHA-DHA inserted into pKCcas9dO by *Hin*dIII and *Spe*I | This work |
| **PCR Primers** | Sequence(5′→ 3′) |  |
| sgRNA-F | TTGG**ACTAGT**TATCCAGGAGGACATGGGGAGTTTTAGAGCTAGAAA | This work |
| sgRNA-R | CTCAAAAAAAGCACCGACTCGG | This work |
| tet-up-F | CCGAGTCGGTGCTTTTTTTGAGACCTTCATCGTCACTCCGTTCT | This work |
| tet-up-R | GCCTGAACCACTGATGAGTCCAACCTCGCCCTGGTTCCAAG | This work |
| tet-down-F | TGGACTCATCAGTGGTTCAGGC | This work |
| tet-down-R | CCC**AAGCTT**GATGTCCTGCGGATCAAGAAGC | This work |
| perm-F | GCA**TCTAGA**TGGACTTCTAGAGCTAGCC | This work |
| perm-R | CGCTTCGCGCATGCCGGTCGACTCTA | This work |
| tetR-F | ACCGGCATGCGAAGTGGTTTGGCGAAGCG | This work |
| tetR-R | CCC**AAGCTT**CCTCCCTCACCACAACGAAT | This work |
| Apr-F | GTCCAATACGAATGGCGAAAAGC | This work |
| Apr-R | ATAACATTCTTCGCATCCCGCC | This work |
| tetR-P-F | GAAGTGGTTTGGCGAAGCG | This work |
| tetR-P-R | CCTCCCTCACCACAACGAAT | This work |
| tetR-H-F | TGGGTCGCGGATCCGAAGGAAGTGGTTTGGCGAAGCG | This work |
| tetR-H-R | CTCGAGTGCGGCCGCACCTCCCTCACCACAACGAAT | This work |

Table S3. Primers, plasmids and strains used in this study

Note: Restriction enzyme sites were italic and bold, overlaping sequences were underlined.

Table S4. qRT-PCR Primers used in this study

|  | **Relative description** | **Sources** |
| --- | --- | --- |
| **qRT-PCR Primers** | Sequence(5′→ 3′) | This work |
| 16s rRNA-F | CGTCAGCTCGTGTCGTGAGA | This work |
| 16s rRNA-R | GTGAAGCCCTGGGCATAAGG | This work |
| *sigF-*F | GCAGATGACCAGCGTGAT | *This work* |
| *sigF-*R | TAGAGGGTGACCACCACG | *This work* |
| *WhiB-*F | GCGACGGACGAAGAACAGG | *This work* |
| *WhiB-*R | CAGCGCGTACTCCAGGCAT | *This work* |
| *WhiA-*F | CCGACGGGCTGAGGTTTC | *This work* |
| *WhiA-*R | GTGCCCGAACAGCTCGTG | *This work* |
| *bldD-*F | TCGTCGGGTCCTATGAGCG | *This work* |
| *bldD-*R | TCACAACTTTGGTGGCAGGC | *This work* |
| *ssgA-F* | CGAGGGCGACGTGACGAT | *This work* |
| *ssgA-R* | AGGTTCTCGTTGCCAGGCAC | *This work* |
| *busA-F* | ACCAACGACGATGAACACGC | *This work* |
| *busA-R* | GCAACCTCCCTGGATTACGG | *This work* |
| *busB-F* | CGCTTGGCTCAGGTGTCGT | *This work* |
| *busB-R* | GAAGACGGATGTTCGTGACCC | *This work* |
| *busC-F* | CAGGGGAACAGGCGAAAT | *This work* |
| *busC-R* | GCGTAGACACGGTTGTTGAG | *This work* |
| *busD-F* | GCAGGGCATTCCGTCCATT | *This work* |
| *busD-R* | ACCGAGCAGGACTTCCAACG | *This work* |
| *busE-F* | GCCTCCACAGCATCCACATC | *This work* |
| *busE-R* | TGTCGGATGCCCGTCGTA | *This work* |
| *busF-F* | ACCAGGTGGACTTCTCGTGC | *This work* |
| *busF-R* | ATCCCGCTGCCTATTTCTCG | *This work* |
| *busG-F* | TCCCGCTCAACCTGTTCCTG | *This work* |
| *busG-R* | CTGCTCATCCGGCAAGCAGA | *This work* |
| *busI-F* | GTCCTTCCATGCCCTGTTTC | *This work* |
| *busI-R* | AGGCCGTCGATCAGTTCTTT | *This work* |
| *busK-F* | ACGACGGCAGCCACATCAAC | *This work* |
| *busK-R* | ACGCCTTCCAGCAGGTTCTT | *This work* |
| *busO-F* | AGCAACTACACGCAGGCACA | *This work* |
| *busO-R* | CCGAGGGTCAACCAGCAGAA | *This work* |
| *busP-F* | TGCGACTGCCTGTGGACTTG | *This work* |
| *busP-R* | TGCCTGTTCCTGGGCTTCTC | *This work* |
| *busS-F* | ACATCTACGACGCGATCCAC | *This work* |
| *busS-R* | TCGCATCGGACAGTTCAAGC | *This work* |
